# Lyme Disease: Call for a “Manhattan Project” to Combat the Epidemic

**DOI:** 10.1371/journal.ppat.1003796

**Published:** 2014-01-02

**Authors:** Raphael B. Stricker, Lorraine Johnson

**Affiliations:** International Lyme and Associated Diseases Society, Bethesda, Maryland, United States of America; The Fox Chase Cancer Center, United States of America

Lyme disease is the most common tick-borne illness in the world today. Until recently, the Centers for Disease Control and Prevention (CDC) reported an average of only 30,000 cases of Lyme disease per year in the United States. Three preliminary CDC studies, however, have indicated that the true incidence of Lyme disease may be greater than 300,000 cases and as high as one million cases per year in the United States. A majority of these cases occur in women and children. Based on this new information, Lyme disease should be recognized as a virulent epidemic that is at least six times more common than HIV/AIDS. In response to these alarming statistics, we review the ongoing problems with diagnosis and treatment of Lyme disease. We propose the need for an HIV/AIDS-style “Manhattan project” to combat this serious epidemic that threatens the physical and mental health of millions of people around the world.

Almost from the moment of its discovery, Lyme disease has been a controversial illness [1], [2]. The disease is caused by a spirochete, *Borrelia burgdorferi*, that is transmitted to humans by the bite of an *Ixodes* tick [3], [4]. Following the discovery in 1982 that a spirochete was the agent of the disease, numerous reports described the protean clinical manifestations of the tick-borne illness, and laboratory testing for the disease was implemented in a haphazard fashion. This poorly directed approach to Lyme disease resulted in the perception in the early 1990s that the disease was being “overdiagnosed and overtreated” [5],[6]. This perception in turn led to a backlash, culminating in the development of stringent diagnostic and therapeutic criteria for Lyme disease [7], [8]. As a result, the diagnosis and treatment of Lyme disease has been limited by a surveillance case definition formulated by the Centers for Disease Control and Prevention (CDC) and supported by the Infectious Diseases Society of America (IDSA) [9], [10]. Use of this stringent case definition has restricted CDC reporting of the disease to about30,000 cases per year in the United States.

In response to this limited view of Lyme disease, a number of practitioners felt that the diagnostic criteria embraced by CDC/IDSA, such as the need for at least five antibodies against a laboratory strain of *B. burgdorferi*, geographical and seasonal restriction of the disease, and the presence of severe objective symptoms, were too restrictive and excluded many sick patients with tick-borne diseases. They also saw treatment failure with the short-term antibiotic regimens recommended by CDC/IDSA guidelines, and they recognized that Lyme disease may be complicated by infection with other tick-borne agents such as *Babesia*, *Anaplasma*, *Ehrlichia*, *Rickettsia*, and *Bartonella*. These practitioners formed the International Lyme and Associated Diseases Society (ILADS), an organization whose viewpoint is that Lyme disease is much more prevalent and complex than the CDC/IDSA criteria allow [11], [12]. Based on the ILADS clinical perspective, it follows that the successful treatment of tick-borne diseases is more challenging than the limited recommendations of CDC/IDSA [3], [12]. The contrasting CDC/IDSA and ILADS perspectives have given rise to the “Lyme Wars,” with sick patients stuck between the “overdiagnosed/overtreated” and “underdiagnosed/undertreated” camps [13],[14]. The controversial nature of Lyme disease, coupled with the relatively small number of cases reported by the CDC, has stunted progress in combatting this illness.

All of this has suddenly changed. Three preliminary reports from the CDC have drastically altered our view of Lyme disease [15]–[17]. The first report, by Hinckley et al., examined laboratory test results for Lyme disease in 2008. The authors concluded that the true annual rate of diagnosed Lyme disease was on the order of 312,000 cases, more than ten times the official number reported by the CDC [15]. The second report, by Hook et al., examined self-reported cases of Lyme disease in the years 2009, 2011, and 2012. The study found that in 2012, Lyme disease was diagnosed in 0.3% of respondents during the previous year; extrapolating from a population of >300 million, as many as one million people would have been diagnosed with Lyme disease in that timeframe. Furthermore, 42% of Lyme disease patients remained ill after 6 months, 12% were ill for more than 3 years, and 36% were treated with antibiotics for more than 8 weeks. Based on these results, the authors concluded that “a very large number of individuals in the US have been diagnosed with Lyme disease” [16].

The third study, by Nelson et al., analyzed private insurance claims related to Lyme disease between 2001 and 2010. The study found that a large number of Lyme disease cases not formally reported are nevertheless diagnosed and treated by healthcare providers. Among inpatients, children aged 5–9 years had the highest rate of Lyme disease diagnosis, while women were diagnosed with Lyme disease more often than men in the outpatient setting. The reason for this gender discrepancy is unclear [17]. In summary, these CDC studies indicate that Lyme disease is far more prevalent than official reporting statistics indicate, with at least 300,000 new Lyme disease cases and as many as one million cases (a majority of them women and children) diagnosed each year in the United States. In comparison to HIV/AIDS, which is diagnosed at a rate of 55,000 new cases per year, Lyme disease appears to be at least six times more common in the United States.

How did Lyme disease reach these epidemic proportions? Three major factors play a role. First, the clinical diagnostic criteria for Lyme disease are too stringent, with only objective signs of the disease, such as an erythema migrans rash, arthritis, meningitis, or carditis, considered relevant [7]. Many patients with late Lyme disease (up to 90%) will only display subjective symptoms of the disease such as fatigue, musculoskeletal pain, and neurocognitive problems, and these patients will often fail to be diagnosed and treated for the disease [3], [18]. Second, commercial laboratory testing for Lyme disease yields poor results, with a sensitivity of only 46% in patients who have been infected for more than 4–6 weeks. Thus, these tests miss more than half of Lyme disease cases [4], [19]. In contrast, testing for HIV/AIDS has a sensitivity of >99.5% and misses less than one in 200 HIV/AIDS cases [3], [4]. Third, treatment for Lyme disease is very restrictive, with CDC/IDSA clinging to the use of monotherapy with questionably effective short courses of antibiotics. This outdated approach contrasts with other serious infectious diseases such as tuberculosis and HIV/AIDS, where long-term combination antimicrobial therapy is the norm [4], [20].

Controversy also persists over the existence of chronic Lyme disease due to persistent infection with *B. burgdorferi* in patients who are untreated or undertreated for the spirochetal illness [8], [21], [22]. While some researchers continue to insist that there is no “credible scientific evidence” for chronic Lyme disease, a growing body of clinical and research evidence supports persistent symptomatic infection with the Lyme spirochete [21]–[24]. Recognition of the 10-fold greater magnitude of the Lyme disease epidemic and persistence of symptoms for more than 3 years supports the concept of chronic Lyme disease [15], [16]. It remains to be determined how the chronic form of the disease is related to the ability of *B. burgdorferi* to penetrate tissue sites, evade the immune response and survive antibiotic therapy.

What needs to be done to combat the growing Lyme disease epidemic? As suggested by others [25], we need to establish a “Manhattan project” along the lines of the approach to the HIV/AIDS epidemic. First, an inclusive panel of clinicians, researchers, patients, and government officials should be established to determine the new approach to Lyme disease using the type of panel balancing recommended by government guidelines for controversial diseases [26]. This panel should revoke the archaic and ineffective IDSA Lyme guidelines and establish new clinical parameters for Lyme disease diagnosis [27], [28]. Second, a uniform standard for Lyme disease testing should be established, with emphasis on a “gold standard” culture and/or PCR test for the spirochete [29]–[31]. This effort would mirror the government-supervised approach to HIV/AIDS that was used to ensure high test sensitivity for that disease, as described above [3], [4]. Third, further trials of antibiotic therapy should be conducted once the “gold standard” testing is in place, with emphasis on combination antimicrobial therapy and encouragement of pharmaceutical industry participation [4], [32], [33]. Although development of a safe and effective Lyme vaccine would be desirable, the failure of a previous Lyme vaccine and the inability over 30 years to develop an effective HIV/AIDS vaccine should serve as a cautionary note that vaccine-based prevention of Lyme disease may not be feasible in the near future [34]–[36].

In summary, preliminary studies from the CDC indicate that the Lyme disease epidemic has reached an unprecedented level with at least 300,000 people and as many as one million people, a majority of them women and children, diagnosed with Lyme disease each year in the United States. The staggering magnitude of the epidemic should prompt the CDC to show leadership in developing new guidelines for the diagnosis and treatment of Lyme disease. A coordinated “Manhattan project” similar to the attack mounted against the HIV/AIDS epidemic is urgently needed to address the serious worldwide threat of Lyme disease.

## References

[1] Harvey WT, Salvato P (2003) ‘Lyme disease’: ancient engine of an unrecognized borreliosis pandemic? Med Hypotheses 60: 742–759. 12710914 10.1016/s0306-9877(03)00060-4

[2] Johnson L, Stricker RB (2004) Treatment of Lyme disease: a medicolegal assessment. Expert Rev Anti Infect Ther 2: 533–557. 15482219 10.1586/14787210.2.4.533

[3] Stricker RB, Johnson L (2011) Lyme disease: The next decade. Infect Drug Resist 4: 1–9. 21694904 10.2147/IDR.S15653 PMC3108755

[4] Stricker RB, Johnson L (2010) Lyme disease diagnosis and treatment: Lessons from the AIDS epidemic. Minerva Med 101: 419–425. 21196901

[5] Steere AC, Taylor E, McHugh GL, Logigian EL (1993) The overdiagnosis of Lyme disease. JAMA 269: 1812–1816. 8459513

[6] Dressler F, Whalen JA, Reinhardt BN, Steere AC (1993) Western blotting in the serodiagnosis of Lyme disease. J Infect Dis 167: 392–400. 8380611 10.1093/infdis/167.2.392

[7] Wormser GP, Dattwyler RJ, Shapiro ED, et al (2006) The clinical assessment, treatment, and prevention of Lyme disease, Human Granulocytic Anaplasmosis, and Babesiosis: Clinical practice guidelines by the Infectious Diseases Society of America. Clin Infect Dis 41: 1089–1134. 10.1086/508667 17029130

[8] Feder HM Jr, Johnson BJ, O'Connell S, Shapiro ED, Steere AC, Wormser GP (2007) Ad Hoc International Lyme Disease Group (2007) A critical appraisal of ‘chronic Lyme disease’. N Engl J Med 357: 1422–30. 17914043 10.1056/NEJMra072023

[9] Johnson L, Aylward A, Stricker RB (2011) Healthcare access and burden of care for patients with Lyme disease: a large United States survey. Health Policy 102: 64–71. 21676482 10.1016/j.healthpol.2011.05.007

[10] Stricker RB, Johnson L (2011) The pain of chronic Lyme disease: moving the discourse backward? FASEB J 25: 4085–7. 22131364 10.1096/fj.11-1203LTR

[11] Burrascano JJ (1993) The overdiagnosis of Lyme disease. JAMA 270: 2682. 10.1001/jama.1993.03510220038016 8192761

[12] Cameron D, Gaito A, Harris N, et al (2004) Evidence-based guidelines for the management of Lyme disease. Expert Rev Anti-Infect Ther 2 1 Suppl: S1–13. 15581390 10.1586/14789072.2.1.s1

[13] Stricker RB, Lautin A (2003) The Lyme Wars: time to listen. Expert Opin Investig Drugs 12: 1609–1614. 10.1517/13543784.12.10.1609 14519082

[14] Kullberg BJ, Berende A, van der Meer JW (2011) The challenge of Lyme disease: tired of the Lyme wars. Neth J Med 69: 98–100. 21444933

[15] Hinckley A, Connally N, Meek J, Johnson BJ, Kemperman M, et al. (2013) TickNET: A survey of testing practices for Lyme disease by large commercial laboratories –United States, 2008. Presented at the 13th International Conference on Lyme Borreliosis and other Tick-Borne Diseases, Boston, MA, August 19, 2013. Available: http://www.poughkeepsiejournal.com/assets/pdf/BK211782914.pdf.

[16] Hook S, Nelson C, Mead P (2013) Self-reported Lyme disease diagnosis, treatment, and recovery: Results from 2009, 2011, & 2012 HealthStyles nationwide surveys. Presented at the 13th International Conference on Lyme Borreliosis and other Tick-Borne Diseases, Boston, MA, August 19, 2013. Available: http://www.poughkeepsiejournal.com/assets/pdf/BK211780914.pdf.

[17] Nelson C, Saha S, Shankar M, Kugeler K, Hinckley A, et al. (2013) Epidemiology and clinical characteristics of Lyme disease diagnosed by health care providers: Results from a large national database study. Presented at the 13th International Conference on Lyme Borreliosis and other Tick-Borne Diseases, Boston, MA, August 19, 2013. Available: http://www.poughkeepsiejournal.com/assets/pdf/BK211781914.pdf.

[18] Cairns V, Godwin J (2005) Post-Lyme borreliosis syndrome: a meta-analysis of reported symptoms. Int J Epidemiol 34: 1340–1345. 16040645 10.1093/ije/dyi129

[19] Phillips SE, Burrascano JJ, Horowitz R, Savely VR, Stricker RB (2006) Lyme disease testing. Lancet Infect Dis 6: 122. 10.1016/S1473-3099(06)70387-5 16500590

[20] Stricker RB (2007) Counterpoint: long-term antibiotic therapy improves persistent symptoms associated with Lyme disease. Clin Infect Dis 45: 149–57. 17578772 10.1086/518853

[21] Berndtson K (2013) Review of evidence for immune evasion and persistent infection in Lyme disease. Int J Gen Med 6: 291–306. 23637552 10.2147/IJGM.S44114 PMC3636972

[22] Stricker RB, Johnson L (2013) Persistent infection in chronic Lyme disease: does form matter? Res J Infect Dis 1: 2.

[23] Miklossy J (2012) Chronic or late Lyme neuroborreliosis: analysis of evidence compared to chronic or late neurosyphilis. Open Neurol J 6: 146–157. 23346260 10.2174/1874205X01206010146 PMC3551238

[24] Ljøstad U, Mygland Å (2013) Chronic Lyme; diagnostic and therapeutic challenges. Acta Neurol Scand Suppl 196: 38–47. 10.1111/ane.12048 23190290

[25] Liegner K (2012) Valley views: Major initiative needed to fight tick-borne infections. Poughkeepsie Journal, August 21, 2012. Available: http://www.poughkeepsiejournal.com/article/20120822/OPINION04/308220005/Valley-Views-Major-initiative-needed-fight-tick-borne-infections.

[26] Viswanathan M, Carey TS, Belinson SE, et al. (2013) Identifying and managing nonfinancial conflicts of interest for systematic reviews. Methods Research Report. AHRQ Publication No. 13-EHC085-EF. Rockville, MD: Agency for Healthcare Research and Quality; May 2013. Available: www.effectivehealthcare.ahrq.gov/reports/final.cfm. 23844449

[27] Johnson L, Stricker RB (2010) The Infectious Diseases Society of America Lyme guidelines: a cautionary tale about development of clinical practice guidelines. Philos Ethics Humanit Med 5: 9. 20529367 10.1186/1747-5341-5-9 PMC2901226

[28] Johnson L, Stricker RB (2010) Final report of the Lyme Disease Review Panel of the Infectious Diseases Society of America: A Pyrrhic victory? Clin Infect Dis 51: 1108–1109. 20925511 10.1086/656690

[29] Sapi E, Kaur N, Anyanwu S, Luecke DF, Datar A, et al (2011) Evaluation of in-vitro antibiotic susceptibility of different morphological forms of Borrelia burgdorferi. Infect Drug Resist 4: 97–113. 21753890 10.2147/IDR.S19201 PMC3132871

[30] Sapi E, Pabbati N, Datar A, Davies EM, Rattelle A, et al (2013) Improved culture conditions or the growth and detection of Borrelia from human serum. Int J Med Sci 10: 362–376. 23470960 10.7150/ijms.5698 PMC3590594

[31] Nolte O (2012) Nucleic acid amplification based diagnostic of Lyme (neuro-)borreliosis - Lost in the jungle of methods, targets, and assays? Open Neurol J 6: 129–139. 23230454 10.2174/1874205X01206010129 PMC3514706

[32] Stricker RB, Delong AK, Green CL, Savely VR, Chamallas SN, et al (2011) Benefit of intravenous antibiotic therapy in patients referred for treatment of neurologic Lyme disease. Int J Gen Med 4: 639–646. 21941449 10.2147/IJGM.S23829 PMC3177589

[33] Donta ST (2012) Issues in the diagnosis and treatment of Lyme disease. Open Neurol J 6: 140–145. 23248715 10.2174/1874205X01206010140 PMC3520031

[34] Stricker RB (2008) Lymerix® risks revisited. Microbe 3: 1–2.

[35] Marks DH (2011) Neurological complications of vaccination with outer surface protein A (OspA). Int J Risk Saf Med 23: 89–96. 21673416 10.3233/JRS-2011-0527

[36] Cohen J (2013) AIDS research. More woes for struggling HIV vaccine field. Science 340: 667. 23661729 10.1126/science.340.6133.667

